# Acute Effect of Folic Acid, Betaine, and Serine Supplements on Flow-Mediated Dilation after Methionine Loading: A Randomized Trial

**DOI:** 10.1371/journal.pctr.0010004

**Published:** 2006-05-19

**Authors:** Margreet R Olthof, Michiel L Bots, Martijn B Katan, Petra Verhoef

**Affiliations:** 1 Wageningen Centre for Food Sciences and Wageningen University, Wageningen, Netherlands; 2 Julius Center for Health Sciences and Primary Care, University Medical Center Utrecht, Utrecht, Netherlands

## Abstract

**Objectives:**

We investigated whether reducing post-methionine homocysteine concentrations via various treatments other than folic acid affects vascular function, as measured through flow-mediated dilation (FMD) of the brachial artery. High fasting and post-methionine homocysteine concentrations are associated with cardiovascular disease risk, but homocysteine might be a surrogate marker for low folate status.

**Design:**

This was a randomized, placebo-controlled, double-blind, crossover study.

**Setting:**

The study took place at Wageningen University in Wageningen in the Netherlands.

**Participants:**

Participants were 39 apparently healthy men and women, aged 50–70 y.

**Interventions:**

Participants ingested 10 mg of folic acid, 3 g of betaine, 5 g of serine, and placebo together with an oral methionine load. Each supplement was tested on two different days.

**Outcome Measures:**

On each of the eight treatment days, plasma homocysteine concentrations and FMD were measured before (*t* = 0 h, fasting) and 6 h (*t* = 6 h) after methionine loading.

**Results:**

The mean (± SD) fasting homocysteine concentrations averaged over the eight test days were 9.6 ± 2.1 μmol/l. Mean fasting FMD was 3.1 ± 2.4 FMD%. A methionine load with placebo increased homocysteine concentrations by 17.2 ± 9.3 μmol/l at 6 h after loading, similar to the increase following methionine loading with folic acid. A methionine load together with betaine and with serine increased homocysteine by 10.4 ± 2.8 μmol/l (*p* < 0.001 relative to placebo) and by 12.1 ± 8.2 μmol/l (*p* < 0.001 relative to placebo), respectively. Methionine loading with placebo did not affect FMD, and neither did methionine loading with folic acid, betaine, or serine; differences relative to placebo were +0.7 FMD% (95%CI, −0.6; 1.9), +0.2 FMD% (−1.0; 1.3), and +0.3 FMD% (−0.8; 1.4), respectively.

**Conclusions:**

Experimentally induced acute changes in homocysteine concentrations did not affect FMD in healthy volunteers. This implies that potential adverse effects of high homocysteine concentrations on the cardiovascular system are not mediated through vascular function. However, homocysteine or folate may affect cardiovascular disease risk through other mechanisms.

## Editorial Commentary

**Background:** It is already known from observational studies that people with high concentrations of homocysteine (an amino acid) in the blood are at increased risk of disease involving the heart and blood vessels, known as cardiovascular disease. Some randomized trials have also shown that lowering homocysteine levels decreases the risk of cardiovascular disease, but not all trials show this. The mechanisms linking homocysteine levels and cardiovascular disease are not well understood. Olthof and colleagues wanted to explore further the mechanisms linking homocysteine levels and the risk of cardiovascular disease. The investigators did a trial in healthy volunteers in which homocysteine concentrations were experimentally raised, then lowered, over short periods of time. The volunteers took a methionine supplement (an amino acid that is converted to homocysteine in the body) to raise homocysteine levels and either betaine (a dietary nutrient), serine (an amino acid), or folic acid (a B vitamin) to lower them. During the trial, the researchers then looked at how well the volunteers' arm arteries functioned, as a surrogate for measuring cardiovascular disease risk.

**What this trial shows:** The investigators found that functioning of the volunteers' arteries, as measured through flow-mediated dilation (FMD), was not affected by the changes in homocysteine levels that were brought about in the experiment.

**Strengths and limitations:** Although the number of participants analyzed in the trial was small (*n* = 39), it was large enough to adequately test the researchers' hypothesis. The vast majority of participants received the experimental treatment, with only one participant dropping out of the study. However, the observations were made over a short period of time, within 6 h of the experimental treatments being given. The investigators did not look at clinical outcomes, such as heart disease or stroke. Therefore, this trial does not provide evidence on whether altered homocysteine levels cause, or could be manipulated to prevent, such clinical outcomes.

**Contribution to the evidence:** This trial adds information on the short-term effects of changes in homocysteine levels.

## INTRODUCTION

High plasma total homocysteine concentrations are associated with higher risk of cardiovascular disease [1,2]. Both fasting homocysteine and the increase of homocysteine concentrations after a methionine-loading test predict the risk of cardiovascular disease [3,4]. However, it remains uncertain whether a high concentration of homocysteine itself or whether a low folate status—its main determinant—is involved in the pathogenesis of cardiovascular disease [5]. Some clinical trials of homocysteine lowering through B-vitamin treatment support the hypothesis that homocysteine is a culprit factor in cardiovascular disease [6,7], but others do not [8–12]. To shed light on this issue, we assessed whether supplementation with homocysteine-lowering nutrients, e.g., folic acid, betaine, and serine, similarly affect the risk of cardiovascular disease.

Betaine is a methyl donor for remethylation of homocysteine into methionine. Serine is the methyl donor for formation of 5-methyltetrahydrofolate from tetrahydrofolate. 5-Methyltetrahydrofolate is subsequently used in remethylation of homocysteine into methionine. Furthermore, in the first step of transsulfuration of homocysteine to cysteine, serine condenses with homocysteine to form cystathionine [13]. We previously showed that a single dose of betaine and of serine together with a methionine load reduces the increase in plasma homocysteine concentrations following a methionine load [14–16].

High homocysteine concentrations might increase risk of cardiovascular disease through impairment of vascular function, an early marker of the atherosclerotic process. Vascular function can be assessed noninvasively through flow-mediated dilation (FMD) of the brachial artery [17,18]. FMD is considered a good alternative for assessing cardiovascular disease risk. FMD is associated with endothelial function in coronary arteries of patients [19,20]. Both coronary endothelial function [21,22] and FMD [23–28] are associated with increased mortality and morbidity risk in patients, as well as in low-risk populations. Patients with hyperhomocysteinemia have impaired vascular function relative to healthy controls [29–32]. In addition, methionine-induced hyperhomocysteinemia in healthy participants also has been shown to lead to impairment in vascular function [33–43].

One study showed that a high dose of 20 mg of folic acid prevents the impairment in FMD following methionine loading, whereas this same dosage did not affect the increase in plasma homocysteine following methionine loading [33]. On the other hand, another study showed no effect on FMD of a 5-mg folic acid dose together with methionine loading [34]. Furthermore, supplementation with folic acid or 5-methyltetrahydrofolate, the naturally circulating form of folate, without methionine loading has been shown to acutely improve vascular function in patients with coronary artery disease [44,45], in hypercholesterolemic patients [46], and in patients with diabetes type II [47]. These studies suggest that folic acid affects FMD through mechanisms largely independent of homocysteine lowering [45]. There are no data on effects of betaine and serine supplementation together with methionine loading on vascular function.

In an attempt to identify whether or not homocysteine itself is involved in cardiovascular disease etiology, we tested the effect of the homocysteine-lowering supplements folic acid, betaine, and serine together with methionine loading on vascular function, as measured through FMD, in healthy participants.

## METHODS

### Participants

Participants were recruited from the pool of volunteers registered at Wageningen University. Eligible volunteers were healthy as assessed by routine medical screening and a general health questionnaire; were between 50 and 70 y old; had a plasma total homocysteine concentration below 26 μmol/l; had no history of cardiovascular disease; had no hypertension; and had not used vitamin B supplements more than once a week in the 3 mo before entering the study. Of those volunteers, 99 participants were assessed for eligibility. Out of 69 eligible participants, 40 participants (19 males) with the highest plasma total homocysteine concentrations were included in this placebo-controlled, double-blind, crossover study (Table 1). The study was conducted at the Division of Human Nutrition, Wageningen University (Wageningen, the Netherlands). The local medical ethics committee approved the protocol, and all volunteers gave their written informed consent.

**Table 1:**
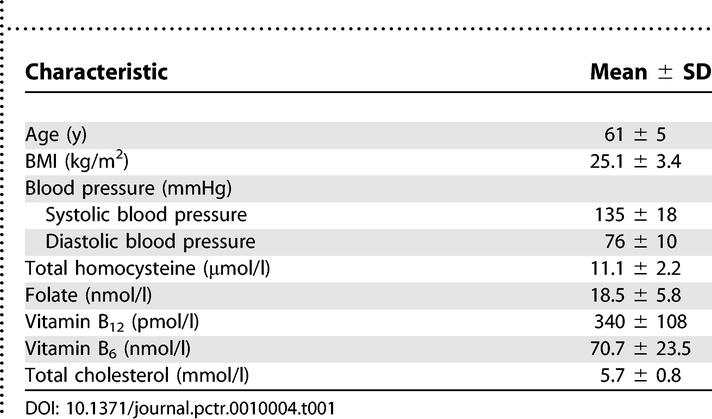
Participant Characteristics at Screening (*n* = 40)

### Interventions

We tested four supplements together with a methionine-loading test (50 mg/kg body weight; all supplements provided by BUFA B.V. Pharmaceutical Products, Uitgeest, the Netherlands). The supplements were (a) 5 g of lactose (placebo); (b) 10 mg of folic acid mixed with 5 g of lactose; (c) 3 g of betaine mixed with 2 g of lactose; and (d) 5 g of l-serine. For each participant, we measured the effects of each supplement on two separate days to get a more precise estimate. Participants came to the university on 8 d divided over 4 wk (two different days per week, i.e., Monday and Wednesday or Tuesday and Thursday). Participants were randomly assigned to a unique treatment order. They received four supplements together with a methionine load, each on a different day in random order spread over the first 2 wk. Subsequently, they again received all four supplements in random order on four different days spread over the second period of 2 wk. There was always at least 1 wk in between the repeat treatments with the same supplements. The study supplements were dissolved in yoghurt or custard and ingested together with the methionine. We chose doses of 3 g of betaine and 5 g of serine because we found in previous studies that these doses considerably lower plasma homocysteine concentrations following a methionine load (by 23% and 37%, respectively) [15,16].

### Objectives

We investigated whether reducing post-methionine homocysteine concentrations via folic acid, betaine, and serine treatment affects vascular function, as measured through FMD in healthy volunteers.

### Outcomes

FMD and plasma homocysteine concentrations were the primary outcome measures. Serum concentrations of vitamins B_12_ and folate were secondary outcome measures.

#### FMD.

Brachial artery measurements were done in participants following an overnight fast on each of the eight treatment days. On these days, FMD was measured before (*t* = 0 h, fasting) and 6 h (*t* = 6 h) after methionine loading. The within-participant coefficient of variation (CV = {SD/mean} × 100%) for FMD was 57% in our study, and this corresponded well to previous measurements in our laboratory [48,49].

At the start of each measurement day, participants rested on a bed for 15 min in a temperature-controlled room (20^ o^C–25 °C). Subsequently, we measured FMD of the brachial artery using a 7.5 MHz linear-array transducer of an ATL Ultramark 9 HDI duplex scanner (Philips Medical Systems, Bothell, Washington, United States). The measurements were done at the brachial artery of the right arm, at the site of the antecubital crease, with an inflatable cuff around the forearm. Arm and ultrasound transducer were held in position with a specially designed fixture (TAF, developed by Meijer, Vascular Imaging Center, Julius Center for Health Sciences and Primary Care, University Medical Centre, Utrecht, the Netherlands) [49]. We chose a segment of the artery of at least 10 mm in length with clear lumen and distinctive vessel walls. All images were 10× zoomed and electronically focused. We first obtained an optimal two-dimensional B-mode ultrasound image of the brachial artery at rest and recorded three baseline images to measure baseline diameter. We then either inflated the cuff around the lower arm to a pressure of 200 mmHg or we inflated the cuff 50 mmHg above systolic blood pressure in cases in which the systolic blood pressure was >150 mmHg. The pressure was kept constant for 5 min to induce ischemia in the forearm and hand, and then the cuff was deflated and image recording was started. In the next 5 min, images of the brachial artery were frozen every 15 s. All measurements were done at the end-diastole by the use of the R-wave of the electrocardiogram. All images were recorded on super-VHS videotape for offline analysis.

The offline reading of ultrasound examinations was done using Brachial Tools, Version 3.2.6 (Medical Imaging Applications, Coralville, Iowa, United States) as has been described in detail elsewhere [49]. One reader, who was unaware of treatment allocation, read all images at the Vascular Imaging Centre of the University Medical Center Utrecht. Each scan was read in duplicate, to limit reading variation. The coefficient of variation (CV = {SD/mean} × 100%) in calculated FMD% between readings was 27%. The reader traced the trailing edge of the adventitia–media interface at the near wall and the leading edge of the media–adventitia interface at the far wall of the brachial artery over a length of at least 3 mm. The distance between these interfaces reflects the lumen diameter. FMD was computed as the percent increase in arterial diameter: FMD% = {(maximum minus baseline)/baseline} × 100%. For reasons of clarity we use “FMD%” as a unit of FMD measurements.

#### Blood sampling and laboratory analyses.

Venous blood was taken from the antecubital vein following an overnight fast and 6 h following methionine loading on each treatment day. Blood samples for analysis of total homocysteine were collected in vacutainer tubes containing EDTA. Samples were mixed and put on ice immediately after collection. Within 30 min, samples were centrifuged for 20 min at 2000 × *g* at 4 °C. For analyses of vitamins B_12_ and folate, blood was collected in vacutainer tubes containing clot activator and a gel to separate serum and cells. About 30 min after blood was collected, samples were centrifuged for 15 min at 2000 × *g* at 4 °C. All samples were stored below −70 °C. Samples were coded to hide the identity and treatment of participants. All samples obtained from one participant were analyzed in the same run.

Total homocysteine concentrations (the sum of all oxidized and reduced forms of homocysteine) were measured by high performance liquid chromatography with fluorescence detection [50]. Serum folate and vitamin B_12_ were measured using a commercial chemiluminescent immunoassay system (IMMULITE 2000, Diagnostic Products Corporation, Los Angeles, California, United States) [51].

#### Standardization procedures.

During the study, participants were not allowed to consume supplements containing B vitamins, antioxidant vitamins (A, beta-carotene, C, and E), or n-3 fatty acids/fish oil supplements, and they were instructed to maintain their physical activity level, dietary habits, and smoking habits during the study.

Participants ate their self-selected diets, except on the days prior to every measurement day and during each measurement day when they visited the university. On these days, we provided the participants with standardized breakfast, lunch, dinner, and snacks. Participants prepared and ate the foods at home on the day prior to the measurement day. Coffee and tea consumption and smoking were not allowed after 6 p.m., and dinner had to be consumed before 8 p.m. From 10 p.m. until after the fasting measurements the next morning (blood sampling, FMD, and blood pressure), participants were not allowed to smoke, eat, or drink anything except for water. The next morning, participants came to the university for measurements, and they received a standardized breakfast together with the methionine load and the supplement, and a lunch and snacks until the measurements were finished (~6 h after methionine loading). After the measurements, participants were free to eat their own foods.

All foods that we provided consisted of normal food products, but foods rich in protein, folic acid, betaine, or choline were avoided. Participants were not allowed to consume any of their own foods during these days, except for coffee and tea. The first time that participants received the standardized foods, they could choose the amounts they wanted to eat of the foods we provided and of the coffee and tea. On all following standardized days, participants received all foods in amounts similar to what they had consumed the first time, and they were instructed to consume the same amounts of coffee and tea as they did the first time. Participants were instructed to eat everything that we provided and not to eat anything else.

### Sample Size

We calculated that 30 participants would be required to detect an absolute difference of 2 FMD% relative to placebo, assuming the within-person standard deviation of the difference in FMD is ~3.96 FMD% [48] (power = 0.8, α = 0.05). We included 40 participants in our study, anticipating that some participants might withdraw from the study. The difference of 2 FMD% we chose for the power calculations is also used for power calculations in other studies [52–54], and in our lab de Roos et al. [55] showed that replacement of dietary saturated fatty acids by trans fatty acids impaired FMD by 1.8 FMD%. Therefore, we assumed that a difference of 2 FMD% in our studies would be plausible. In addition, a methionine-loading test increases plasma homocysteine concentrations drastically and can completely abolish FMD [56]. We assumed that betaine and serine supplements would largely prevent the impairment in FMD following methionine loading, because our previous studies have shown that betaine and serine supplementation together with methionine loading drastically reduce post-methionine-loading homocysteine concentrations by 30%–40% [15,16]. Folic acid supplementation can also prevent the impairment in FMD following methionine loading, despite the fact that folic acid does not affect post-methionine-loading homocysteine concentrations [33].

### Randomization Procedures

A person not further involved in the study assigned codes to the study treatments and randomly allocated the selected participants to a unique treatment order and kept the key in a sealed envelope. The participants and all others involved in this study were unaware of treatment allocation. The principal investigator performed unblinding of the treatment allocation after the study had ended and laboratory analyses were complete.

### Statistical Analysis

For each FMD measurement, we averaged the duplicate readings of the ultrasound images. For FMD, plasma homocysteine, serum folate, and vitamin B_12_ concentrations, we calculated per participant and per measurement day the difference between fasting values and values at 6 h after methionine loading. Subsequently, we calculated the mean of the duplicate measurements per participant for each treatment. We compared the treatments' effects on FMD, plasma homocysteine concentrations, and B-vitamin concentrations with analysis of variance with repeated measurements (following the General Linear Models procedure in SPSS, Version 12.0). If the analysis of variance indicated a statistically significant overall treatment effect, comparisons of means were performed using the Bonferroni correction, and 95% confidence intervals (CI) corresponding with the differences in means between the treatments were calculated. The 95%CI for the differences in means between fasting and 6 h following methionine loading (Table 2) were calculated from the observed differences assuming a normal distribution. We did not test for carryover effects since every participant had a unique treatment order. Moreover, we did not expect carryover effects in our study. Previous studies have shown that the effects on homocysteine concentrations of methionine loading alone [33] or with betaine [14] or with serine [16] all return to baseline within 24 h. Folic acid does not affect homocysteine concentrations following methionine loading, but effects of folic acid supplementation on FMD also return to baseline within 24 h [33]. For safety reasons, we always planned at least 2 d washout in between the measurement days.

**Table 2:**
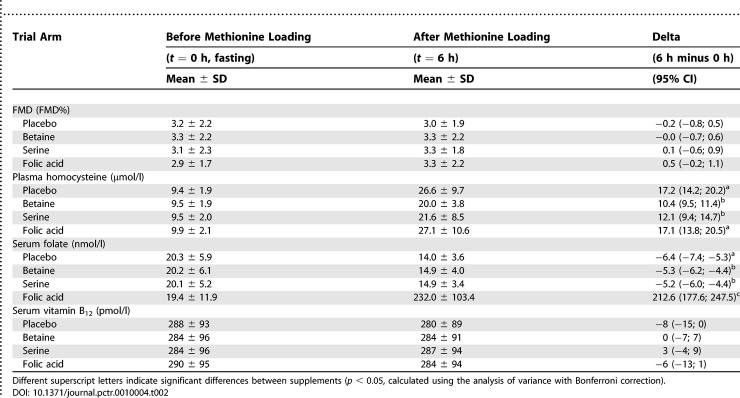
FMD, Concentrations of Total Plasma Homocysteine, Concentrations of Folate and Vitamin B_12_ in 39 Participants Who Ingested Placebo, 3 g of Betaine, 5 g of Serine, and 10 mg of Folic Acid together with a Methionine-Loading Test (50 mg of Methionine per Kilogram of Body Weight) in Random Order, in Crossover Design

All statistical analyses were performed with SPSS, Version 12.0.

## RESULTS

### Participant Flow

**Figure 1:**
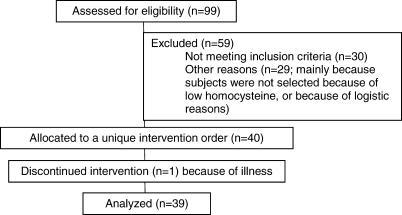
Participant Flow and Recruitment Flow Chart

Participant flow is show in Figure 1. Volunteers were recruited from June to September 2002. The intervention started August 2002 and was completed in July 2003.

### Baseline Data

Participant characteristics at screening are shown in Table 1.

### Numbers Analyzed

Of those who were enrolled, 39 participants completed the study. One male participant withdrew due to illness not related to the study.

### Outcomes

Methionine loading with placebo increased mean plasma homocysteine concentrations from 9.4 μmol/l at baseline to 26.6 μmol/l at 6 h following methionine loading (Table 2). Betaine reduced this increase in homocysteine concentrations by 40% and serine by 30%, whereas folic acid did not reduce the increase in plasma homocysteine concentrations following methionine loading (Table 2).

**Figure 2:**
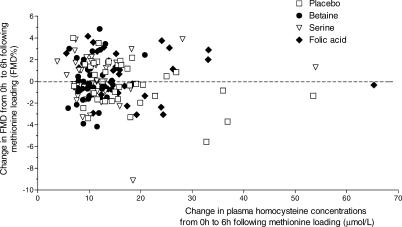
Individual Responses in Plasma Homocysteine Concentrations Shown are the individual responses in plasma homocysteine concentrations (in micromoles per liter) from 0 h to 6 h following methionine loading versus the responses in FMD (FMD%) from 0 h to 6 h following methionine loading in 39 participants who ingested placebo, 3 g of betaine, 5 g of serine, and 10 mg of folic acid together with a methionine-loading test (50 mg of methionine per kilogram of body weight) in random order in a crossover design. Each mark represents the mean of FMD measurements on two different days in one participant.

FMD was not affected by methionine loading with placebo, nor by combinations of methionine with betaine, with serine, or with folic acid (Table 2; Figure 2). Differences in FMD relative to placebo were +0.2 FMD% (−1.0; 1.3) following methionine loading with betaine, +0.3 FMD% (−0.8; 1.4) following methionine loading with serine, and +0.7 FMD% (95%CI, −0.6; 1.9) following methionine loading with folic acid. The mean (± SD) fasting baseline diameter of the brachial artery prior to methionine loading together with placebo, betaine, serine, and folic acid supplementation was 4.23 ± 0.75 mm, 4.22 ± 0.79 mm, 4.25 ± 0.75 mm, and 4.18 ± 0.77 mm, respectively. At 6 h following methionine loading together with placebo, betaine, serine, and folic acid supplementation, mean baseline diameter was 4.28 ± 0.78 mm, 4.27 ± 0.85 mm, 4.27 ± 0.73 mm, and 4.29 ± 0.76 mm, respectively. Fasting mean baseline diameter of the brachial artery as well as the difference between fasting and post-methionine-loading baseline diameters was not significantly different between supplements.

Mean serum folate concentrations decreased from 20.3 nmol/l in the fasting state to 14.0 nmol/l at 6 h after methionine loading with placebo. As expected, concentrations of serum folate were increased more than 10-fold after methionine loading together with the folic acid treatment. Methionine loading together with betaine or serine slightly, but significantly, attenuated the decrease in serum folate concentrations seen with placebo. The concentration of vitamin B_12_ was not affected by the treatments (Table 2).

### Adverse Events

No serious adverse events were reported in this study. Non-serious adverse events occurred 30 times in 15 participants. The adverse events were diverse and unlikely related to the treatment. Most commonly occurring events were headache (six times) and diarrhea (ten times, of which nine times were reported by one participant).

## DISCUSSION

### Interpretation

Our study is the first we know to investigate the effects of various homocysteine-lowering supplements on FMD following methionine loading. We found that methionine-induced hyperhomocysteinemia did not impair FMD in healthy elderly volunteers. In addition, we found that the reduction in the increase in plasma homocysteine concentrations following methionine loading, by means of several supplements, did not affect FMD. This suggests that homocysteine, if involved in early pathogenesis of cardiovascular disease, does not exert its action through a mechanism related to vascular function in healthy individuals.

We are confident that we designed and performed our study well.

First, we selected participants with elevated plasma homocysteine concentrations, who are expected to have a greater response to the homocysteine-lowering interventions than randomly selected individuals.

Second, we standardized the FMD measurement to the maximum of what is possible in a free-living situation. In order to reduce the variation in the FMD measurement due to diet, we provided all foods and standardized coffee and tea consumption on the days preceding the FMD measurements and on the mornings of methionine loading until the last FMD measurement that day. We standardized the timing of eating and smoking on the evening before measurements and on the days of the methionine loading. During the entire study we asked the volunteers to keep physical activity, smoking, and dietary patterns as usual. The supplements were ingested under our supervision, and thus noncompliance could not explain the outcome.

Finally, in order to limit the influence of variation in FMD between participants, we chose a crossover design, so that each participant was his or her own control. Furthermore, in order to reduce the within-participant variation of the FMD measurement and the variation in offline analysis of the ultrasound images [48], we performed duplicate FMD measurements in each participant on each treatment and we did the offline analysis in duplicate. The FMD measurement itself was performed similar to previous studies done in our laboratory. In one study FMD was not acutely impaired after test meals with saturated or trans fatty acids [57].

### Overall Evidence and Generalizability

The authors are not aware of published systematic reviews or meta-analyses on the effect of homocysteine lowering following a methionine load on FMD. Most previous studies show that vascular function is impaired after methionine loading [33–43], although some do not [37,58–60]. An explanation for the fact that we did not find an impairment in FMD following methionine loading could be that we used a lower dose of methionine (50 mg of methionine per kilogram of body weight) than most other studies do (100 mg of methionine per kilogram of body weight). We chose this lower dose of methionine because it is more in the physiological range of dietary methionine intake. Moreover, other studies have shown that methionine doses in the range of 10–50 mg/kg effectively increase plasma homocysteine concentrations and impair FMD [56,61]. In conclusion, it seems unlikely that the lower dose of methionine explains the lack of an effect on FMD in our study.

Similar to previous supplementation studies, betaine and serine supplementation together with methionine loading effectively reduced the increase in plasma homocysteine concentrations [14–16]. However, there was no effect on FMD. We are not aware of other published studies that have investigated effects of betaine or serine supplementation on FMD following a methionine load.

Although folic acid has no acute homocysteine-lowering effect following methionine loading [14], previous studies have shown that folic acid might acutely affect vascular function via mechanisms independent from homocysteine lowering [33,44,45]. Contrary to our expectations, FMD was not affected by folic acid treatment in our study. This is in line with one other study in healthy volunteers that finds no acute effect on FMD of coadministration of 5 mg of folic acid together with the methionine load following methionine loading [34]. However, most studies show that supplementation with folic acid together with a methionine load [33] or folic acid [45] or 5-methyltetrahydrofolate [44,46,47] alone can acutely improve vascular function. We would like to raise the point that publication bias might have resulted in underrepresentation of studies that did not find impairment in FMD following a methionine load or of studies that did not find an improvement of FMD following folic acid treatment.

The results of this study are also in line with the findings of our study on chronic effects of homocysteine lowering on FMD after an overnight fast [62].

However, it remains possible that homocysteine affects vascular function in specific high-risk populations. For example, vitamin C improved FMD in homocystinuric patients, but it did not in control participants [30]. Therefore, our study leaves open the possibility that homocysteine is related to vascular function in specific populations, e.g., patients with cardiovascular disease or patients at high risk for cardiovascular disease.

### Study Limitations

We note in passing that fasting homocysteine levels were lower during the trial than during the screening (on average 9.6 versus 11.1 μmol/l). This could be due to regression to the mean or other causes. Whatever the cause, it did not affect our outcomes because it occurred on all treatments. Moreover, we always used the differences between treatments and placebo as our outcomes. Therefore, we do not expect that possible regression to the mean affected the interpretation of our results.

Although we have performed the FMD with utmost care, there are some methodological issues to discuss. First, we did not perform a nitric oxide (NO)-independent vasodilation test, usually done with sublingual nitroglycerine (NTG). The rationale for such a test is to show whether or not the artery is capable of responding to NO given directly to participants (endothelium-independent vasodilation). Since we selected healthy participants in our study, we assumed that all participants would have an FMD response and we therefore did not confirm this with an NTG test. Moreover, treatment effects on the NTG test were not expected [55,63,64]. As we have seen an FMD response in all of our participants, we do not think that the lack of NTG measurements affects the validity of our findings.

Second, we did not perform Doppler measurements after the cuff release. The Doppler measurements reflect the stimulus (i.e., the blood flow) that elicits the FMD response, and through these measurements one may examine whether the differences between participants and between visits are due to differences in exposure to increased blood flow. We did not perform the Doppler measurements in order to optimize the measurements of the B-mode imaging. The time window to switch between B-mode and the Doppler mode in the ultrasound machine was too short to perform valid Doppler measurements and at the same time capture reliable images for lumen diameter measurements within the first period after cuff release, which is the most important phase in which dilation occurs. However, it is highly unlikely that the stimulus will differ between visits, as the measurements were done in a standardized manner, by the same technicians using an identical protocol. Moreover, the FMD was measured in duplicate on each treatment, which minimized variations in the FMD responses that were not due to the treatments. Therefore, the validity of our findings is not severely hampered by the lack of the Doppler measurement.

### CONCLUSION

We showed that changes in plasma homocysteine concentrations through methionine loading with placebo did not acutely affect vascular function in healthy elderly volunteers. Furthermore, methionine loading together with a high dose of folic acid, betaine, or serine did not affect FMD either. This may indicate that the potential adverse effects of high homocysteine on the cardiovascular system are not mediated through changes in vascular function. However, homocysteine or folate may affect cardiovascular disease risk through mechanisms other than impaired vascular function.

## SUPPORTING INFORMATION

CONSORT Checklist Click here for additional data file. (56 KB DOC)

Trial Protocol Click here for additional data file. (142 KB DOC)

Alternative Language Abstract Click here for additional data file. Translation of the abstract into Dutch by Margreet R. Olthof.
(30 KB DOC)

## Abbreviations

CI: confidence interval
CV: coefficient of variation
FMD: flow-mediated dilation
NO: nitric oxide
NTG: nitroglycerine
