# Supplementary material for: Acute Effect of Folic Acid, Betaine, and Serine Supplements on Flow-Mediated Dilation after Methionine Loading: A Randomized Trial
Source: PLoS Clin Trials. 2006 May 19;1(1):e4. doi: 10.1371/journal.pctr.0010004 (PMC1488894; doi:10.1371/journal.pctr.0010004)
Supplement: CONSORT Checklist [file pctr.0010004.sd001.doc]

**CONSORT Checklist**

### Olthof et al.

### Effect of folic acid, betaine and serine supplementation on flow mediated dilation following a methionine load: A randomized controlled trial in healthy elderly volunteers

| **PAPER SECTION And topic** | **Item** | Description | **Reported in section:** |
| --- | --- | --- | --- |
| TITLE & ABSTRACT | 1 | How participants were allocated to interventions (*e.g*., "random allocation", "randomized", or "randomly assigned"). | - Title and abstract. |
| **INTRODUCTION** Background | 2 | Scientific background and explanation of rationale. | - Introduction. |
| METHODS |  |  |  |
| Participants | 3 | Eligibility criteria for participants and the settings and locations where the data were collected. | - Methods, subsection: participants |
| Interventions | 4 | Precise details of the interventions intended for each group and how and when they were actually administered. | - Methods, subsection: interventions |
| Objectives | 5 | Specific objectives and hypotheses. | - Methods, subsection: objectives |
| Outcomes | 6 | Clearly defined primary and secondary outcome measures and, when applicable, any methods used to enhance the quality of measurements (*e.g.*, multiple observations, training of assessors). | - Methods, subsection: outcomes |
| Sample size | 7 | How sample size was determined and, when applicable, explanation of any interim analyses and stopping rules. | - Methods, subsection: sample size |
| Randomization -- Sequence generation | 8 | Method used to generate the random allocation sequence, including details of any restrictions (*e.g*., blocking, stratification) | - Methods, subsection: randomization and blinding |
| Randomization -- Allocation concealment | 9 | Method used to implement the random allocation sequence (*e.g*., numbered containers or central telephone), clarifying whether the sequence was concealed until interventions were assigned. | - Methods, subsection: randomization and blinding |
| Randomization -- Implementation | 10 | Who generated the allocation sequence, who enrolled participants, and who assigned participants to their groups. | - Methods, subsection: randomization and blinding |
| Blinding (masking) | 11 | Whether or not participants, those administering the interventions, and those assessing the outcomes were blinded to group assignment. When relevant, how the success of blinding was evaluated. | - Methods, subsection: randomization and blinding |
| Statistical methods | 12 | Statistical methods used to compare groups for primary outcome(s); Methods for additional analyses, such as subgroup analyses and adjusted analyses. | - Methods, subsection: statistical analysis |
| RESULTS |  |  |  |
| Participant flow | 13 | Flow of participants through each stage (a diagram is strongly recommended). Specifically, for each group report the numbers of participants randomly assigned, receiving intended treatment, completing the study protocol, and analyzed for the primary outcome. Describe protocol deviations from study as planned, together with reasons. | - Figure 1 |
| Recruitment | 14 | Dates defining the periods of recruitment and follow-up. | - Results, subsection: participants and recruitment |
| Baseline data | 15 | Baseline demographic and clinical characteristics of each group. | - Table 1 |
| Numbers analyzed | 16 | Number of participants (denominator) in each group included in each analysis and whether the analysis was by "intention-to-treat". State the results in absolute numbers when feasible (*e.g*., 10/20, not 50%). | - Results, subsection: numbers analyzed - Table 2 - Figure 1 |
| Outcomes and estimation | 17 | For each primary and secondary outcome, a summary of results for each group, and the estimated effect size and its precision (*e.g.*, 95% confidence interval). | - Results, subsection: outcomes - Table 2 - Figure 2 |
| Ancillary analyses | 18 | Address multiplicity by reporting any other analyses performed, including subgroup analyses and adjusted analyses, indicating those pre-specified and those exploratory. | - Results, subsection: outcomes - Table 2 |
| Adverse events | 19 | All important adverse events or side effects in each intervention group. | - Results, subsection: adverse events |
| DISCUSSION |  |  |  |
| Interpretation | 20 | Interpretation of the results, taking into account study hypotheses, sources of potential bias or imprecision and the dangers associated with multiplicity of analyses and outcomes. | - Discussion, subsection: interpretation |
| Generalizability | 21 | Generalizability (external validity) of the trial findings. | - Discussion, subsection: overall evidence and generalizability, and study limitations |
| Overall evidence | 22 | General interpretation of the results in the context of current evidence. | - Discussion, subsection: overall evidence and generalizability, and study limitations |
