# Supplementary material for: Acute Effect of Folic Acid, Betaine, and Serine Supplements on Flow-Mediated Dilation after Methionine Loading: A Randomized Trial
Source: PLoS Clin Trials. 2006 May 19;1(1):e4. doi: 10.1371/journal.pctr.0010004 (PMC1488894; doi:10.1371/journal.pctr.0010004)
Supplement: Alternative Language Abstract [file pctr.0010004.sd003.doc]

### **Abstract S1.** Translation of the abstract into Dutch by Margreet R. Olthof

### **Acuut effect van foliumzuur, betaïne en serine suppletie op vaatwandfunctie na een methionine belasting test: een gerandomiseerde gecontroleerde interventiestudie bij gezonde ouderen**

Margreet R. Olthof, PhD; Michiel L. Bots, MD, PhD; Martijn B. Katan, PhD; Petra Verhoef, PhD

**Doel**

Wij hebben onderzocht of het reduceren van de concentratie homocysteïne na een methionine belasting test met verschillende supplementen, anders dan foliumzuur, de vaatwandfunctie beïnvloedt. Vaatwandfunctie werd gemeten in de armslagader met ‘flow mediated vasodilation’ (FMD), ofwel vaatverwijding door toegenomen bloeddoorstroming. Hoge concentraties homocysteïne in nuchtere toestand en na methionine belasting zijn beiden geassocieerd met het risico van hart-en vaatziekten, maar het is ook mogelijk dat homocysteïne een marker is voor een lage folaatstatus.

**Studieopzet**

Gerandomiseerde, placebo-gecontroleerde, dubbel-blinde, crossover studie.

**Plaats**

Wageningen Universiteit, Wageningen, Nederland

**Deelnemers**

Negenendertig gezonde mannen en vrouwen tussen 50 en 70 jaar.

**Interventies**

Deelnemers namen 10 mg foliumzuur, 3 g betaïne, 5 g serine en placebo in, samen met een methionine belasting test. Elk supplement werd getest op 2 verschillende dagen.

**Uitkomstmaten**

Op elk van de 8 testdagen werd de plasma homocysteïne concentratie en FMD gemeten; voor (t=0 uur, nuchter) en 6 uur na methionine belasting.

**Resultaten**

De gemiddelde (±SD) concentratie nuchter homocysteïne over de 8 testdagen was 9.6 ± 2.1 μmol/L. Gemiddelde nuchtere FMD was 3.1 ± 2.4 FMD%. Een methionine belasting test samen met placebo verhoogde de concentratie homocysteïne met 17.2 ± 9.3 μmol/L 6 uur na methionine belasting; dit was gelijk aan de toename in homocysteïne na methionine belasting samen met foliumzuur. Een methionine belasting test samen met betaïne en serine verhoogde de concentratie homocysteïne met respectievelijk 10.4 ± 2.8 μmol/L (P<0.001 ten opzichte van placebo) en 12.1 ± 8.2 μmol/L (P<0.001 ten opzichte van placebo). Een methionine belasting test samen met placebo had geen effect op FMD. Een methionine belasting test samen met foliumzuur, betaïne of serine had ook geen effect op FMD; de verschillen ten opzichte van placebo waren respectievelijk +0.7 FMD% (95% betrouwbaarheidsinterval: -0.6;1.9); +0.2 FMD% (-1.0;1.3); +0.3 FMD% (-0.8;1.4).

**Conclusie**

Experimenteel geïnduceerde, acute veranderingen in de concentratie homocysteïne hadden geen effect op FMD bij gezonde vrijwilligers. Dit kan betekenen dat mogelijk ongunstige effecten van hoge homocysteïne concentraties op het hart-en vaatsysteem niet werken via vaatwandfunctie. Homocysteïne of folaat zouden mogelijk wel via andere mechanismen dan vaatwandfunctie het risico van hart-en vaatziekten kunnen beïnvloeden.
